# Ureteroarterial Fistula: A Diagnosis Which Is Not Always Black and White

**DOI:** 10.1155/2021/8165991

**Published:** 2021-08-10

**Authors:** A. Haffar, T. Trump, A. A. Elbakry, K. McCluskey, M. W. Salkini, A. Luchey

**Affiliations:** ^1^West Virginia University School of Medicine, West Virginia, USA; ^2^Department of Urology, West Virginia University, West Virginia, USA; ^3^Department of Interventional Radiology, West Virginia University, West Virginia, USA

## Abstract

Ureteroiliac artery fistulas are a rare, life-threatening condition that requires a high index of suspicion for prompt diagnosis. Presurgical diagnosis is challenging as this condition can lie hidden despite advanced imaging modalities. We present two cases of patients presenting with gross hematuria and exsanguination in the setting of a ureteroiliac artery fistula. These cases highlight the difficulties in timely diagnosis and treatment in a multidisciplinary team.

## 1. Introduction

A ureteroiliac artery fistula (UIAF) is a rare, life-threatening condition that can present suddenly with gross hematuria. Diagnosis is often delayed due to its insidious nature. The incidence of this condition has increased, secondary to risk factors such as pelvic radiotherapy, genitourinary surgery, chronic ureteral stenting, and peripheral arterial disease [1]. Despite its rarity, the diagnosis should be highly suspected when assessing patients with such risk factors due to the associated morbidity and mortality. We hereby present two cases of patients presenting with gross hematuria and exsanguination in the setting of a ureteroiliac artery fistula.

## 2. Case Presentation

### 2.1. Case 1

A 55-year-old female with a previous history of pelvic radiation and exenteration for cervical cancer presented with lower abdominal pain and gross hematuria from bilateral nephrostomy tubes and ileal conduit. She had a history of bilateral hydroureteronephrosis managed with chronic percutaneous nephroureteral stents. Of pertinence, she had a history of complicated UTIs, and her right nephroureteral stent was exchanged 2 weeks prior to presentation without complications.

On examination, the patient's abdominal pain was localized to the lower abdomen without radiation. She was noted to be afebrile with a heart rate of 97, respiratory rate of 18, and blood pressure of 129/83 mmHg. Hematuria from both nephrostomy tubes and ileal conduit was noted.

**Figure 1:**
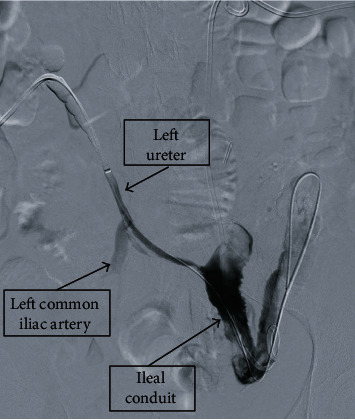
Left nephrostogram with contrast noted extravasating through the fistula tract from the left ureter into the left iliac artery.

**Figure 2:**
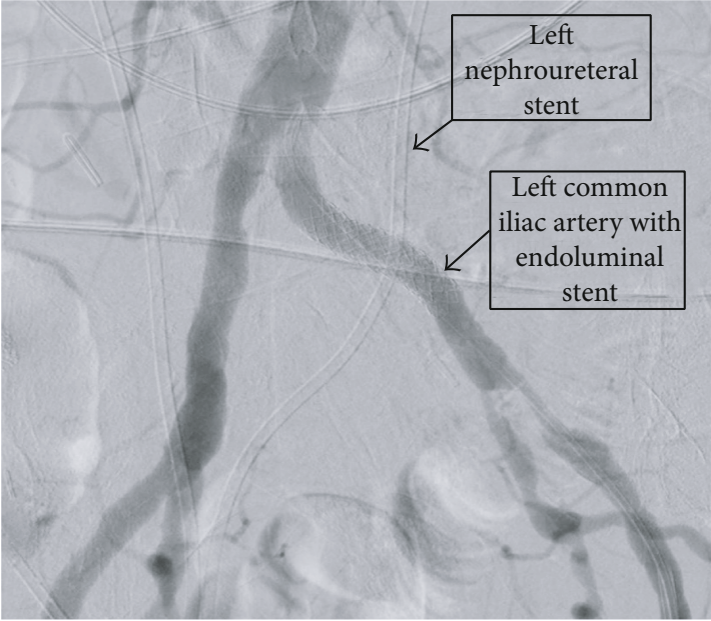
Arteriogram following endograft placement with no contrast exiting the arterial system. Left nephroureteral stent is noted traversing the location of graft placement highlighting the region of fistula formation.

Initial workup was significant for anion gap metabolic acidosis with a pH of 7.10, pCO_2_ of 19 mmHg, and a bicarbonate of 7.2 mEq/L. The patient was anemic with a hemoglobin of 8.1 g/dL. Blood urea nitrogen was found to be 37 mg/dL with a creatinine of 4.38 mg/dL, from a baseline creatinine of 3 mg/dL. The patient was initially managed empirically for a presumed urinary tract infection with intravenous piperacillin/tazobactam. CT imaging from an outside facility was unavailable but reportedly demonstrated an appropriate position of the bilateral nephroureteral stents. Ultrasound indicated bilateral hydronephrosis. Exchange of bilateral nephroureteral stents was scheduled for the following day. Later that evening, her hematuria had progressively worsened, with serial hemoglobin values dropping to 4.2 mg/dL, with associated hemodynamic instability. She was successfully resuscitated and stabilized with 4 units of blood, 1 unit of platelets, 1 unit of FFP, and 1 liter bolus of intravenous fluids. Upon repeat examination, frank hemorrhage was discovered at her left nephroureteral stent and the right nephroureteral stent demonstrated blood mixed with urine; despite this, the patient was asymptomatic. The CT abdomen and pelvis without contrast was performed and was negative. She was then taken for bilateral exchange of nephroureteral stents by interventional radiology. The left stent was removed first, and a significant amount of blood was noted along the stent tract, with an unknown source of the bleeding. Antegrade nephrostogram was performed, and a large fistula between the left ureter and the left common iliac artery was noted (Figure 1). In an attempt to control and tamponade the bleeding, a nephroureteral stent was placed. A rapid decision was made to perform a pelvic angiogram with stenting of the left common iliac artery. An 8 mm × 39 mm VBX covered stent was advanced and deployed in the midcommon iliac artery, with a follow-up angiogram showing no evidence of pseudoaneurysm, active bleeding, or fistula (Figure 2). The patient progressively improved postop with resolution of her acute kidney injury and was discharged 7 days postadmission. As of 6 months postoperatively from this procedure, the patient has been without further complications regarding the fistula. She continues to have regular exchange of her nephroureteral stents and has been without further hematuria.

### 2.2. Case 2

A 73-year-old female presented with shortness of breath, dizziness, and gross hematuria. She had a past medical history of cervical cancer requiring radiation and chemotherapy, chronic kidney disease stage IIIB, and chronic normocytic anemia. The patient has a history of left ureteral stricture with severe hydronephrosis managed with a chronic indwelling metal ureteral stent with regular exchanges for four years. She was initially planned to have a left nephrectomy 1 month prior to her presentation, but it was postponed due to a hemoglobin of 4 g/dl, requiring hospitalization at an outside facility. She reported that her stent was most recently exchanged 5 months prior to her presentation.

On examination, the patient was hemodynamically stable. Frank blood was found in her Foley bag without suprapubic tenderness. Of note, she had hemoglobin of 3.8 g/dL and her creatinine was 1.51 mg/dL. CT imaging with contrast was unable to demonstrate an active source of bleeding but did show severe left hydronephrosis and hydroureter as well as a segmental pulmonary embolus. Subsequent lower extremity Doppler imaging revealed multiple deep venous thrombi. She was initially managed with blood transfusions and continuous bladder irrigation. Interventional radiology was consulted for the placement of an IVC filter in the setting of pulmonary embolus with active bleeding prohibiting the start of anticoagulation. Arteriogram was done during the placement of the IVC filter but failed to demonstrate any evidence of ureterovascular fistula.

Two days later, after continued gross hematuria and sustained anemia, the patient was taken to the OR for cystourethroscopy with left retrograde pyelogram and left ureteral stent exchange. Cystoscopy did not reveal any obvious masses or lesions, but active bleeding was noted from the left ureteral orifice after removal of the ureteral stent. Subsequent left retrograde pyelogram did not reveal any obvious source for continued hematuria; in particular, this was negative for any obvious fistula involving the ureter. The left ureter was markedly dilated, and there were multiple large filling defects, likely clots from the upper tract bleed. A left 6 French × 24 cm Double-J silicone stent was placed under direct visualization. The decision was made to perform left renal artery arteriogram and left lumbar and pelvic angiography which again showed no renal vascular abnormalities or arterioureteral fistula. A decision was then made to embolize the left renal artery. Despite embolization, the patient still had continuous hematuria requiring CBI. Due to continued suspicion for ureteroiliac fistula, a decision was made to proceed with definitive management and perform robotic nephroureterectomy with excision of the ureteroiliac fistula.

**Figure 3:**
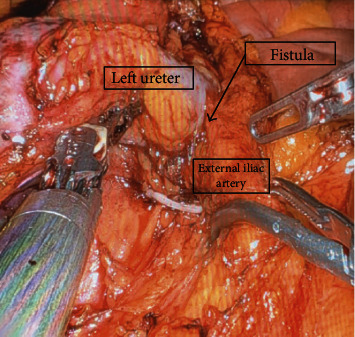
Left ureter raised with left bipolar forceps and small Hem-o-lok clip controlling the divided smaller vascular connection with a laparoscopic Satinsky clamp placed on the left external iliac artery proximal to the ureteroiliac fistula.

The patient was taken to the operating room and positioned in a modified right lateral decubitus position. The procedure proceeded accordingly, and nephrectomy was completed uneventfully. At the stage of dissecting the ureter, it began to pulsate, indicating a possible ureteroiliac fistula. A small vessel directly communicating with the ureter and the left external iliac artery was identified and clipped with a Hem-o-lok and divided with good hemostasis (Figure 3). A much larger vessel was then identified inferiorly. An additional assistant port was placed, and a laparoscopic Satinsky clamp was used to clamp the external iliac artery. An attempt was made to dissect and divide the vascular connection with the ureter, but brisk bleeding was encountered. This proved to be very difficult to control robotically, and the decision was made to convert to open repair using a modified Gibson incision. Heavy bleeding was encountered, and the left common iliac artery was subsequently clamped which controlled active bleeding temporarily. The ureter was separated from the external iliac artery at the level of the fistula leaving a large defect of the fistula in the wall of the external iliac artery. Following temporization of the bleeding, vascular surgery was consulted, and they were able to suture the arteriotomy. With hemostasis maintained, the dissection of the distal left ureter was continued and clipped at the ureterovesical junction. A total of 8 units of packed red blood cells and 2 units of FFP were given with an estimated blood loss of 2500 mL.

Later that day, the patient was discovered to have faint left popliteal arterial pulse by Doppler with no pulse distally. The patient was taken back to the OR with vascular surgery for angiogram of the left lower extremity due to acute limb ischemia. Acute thrombosis was identified, and thrombectomy of the left common iliac artery and external iliac artery was performed, along with balloon angioplasty of the left common iliac artery and external iliac artery. A four-compartment fasciotomy was subsequently performed. During the course of her hospitalization, a total of 19 units of packed red blood cells, 4 units of FFP, and 2 units of cryoprecipitate were given. The patient progressively improved with resolution of her hematuria and was discharged to an inpatient rehabilitation facility. As of 4 months postoperatively, the patient has been without any adverse events. She had her IVC filter removed and was started on therapeutic anticoagulation without development of hematuria.

## 3. Discussion

A ureteroiliac artery fistula in the setting of ileal conduit is an exceedingly rare condition. They often occur in the setting of prior genitourinary surgery, chronic ureteral stenting, radiation therapy, and prior vascular pathology [2]. Additionally, pelvic lymphadenectomy has been proposed to render the vasculature more susceptible to fistula formation [3]. Despite the possibly lethal nature of this condition, the presenting symptoms are often innocuous. Although hematuria is the most common presenting symptom, and flank pain, urinary retention, and infection have also been described [4]. Ultimately, in a patient with a complicated genitourinary tract surgery/history presenting with hematuria and other equivocal symptoms, the physician's index of suspicion for UIAF should be heightened. Early involvement of vascular surgery/interventional radiology in the management is of paramount importance.

The principal difficulty in treating UIAF is diagnosing the condition in a timely manner, as no imaging modality is sensitive enough. CT, commonly done emergently in response to gross hematuria, has very low sensitivity and is rarely diagnostic [5]. Ureteroscopy can be performed and demonstrate pulsatile blood flow; however, in the setting of brisk bleeding, visualization could be difficult [5]. Angiography has even less sensitivity, as low as 23%-41% [6]. However, angiography with concurrent manipulation of a chronic nephroureteral stent was shown to improve sensitivity to 100% at one institution [6]. The sensitivity of provocative retrograde pyelogram was shown to be as low as 63%, which was demonstrated by our second case [7]. Ultimately, no single test will be perfectly diagnostic and will likely need to be reperformed.

Prior to advancements in endovascular techniques, open surgical repair was the predominant manner in which UIAF was treated and was considered the gold standard [8, 9]. However, this was often challenging as these patients have a complex history of genitourinary surgery, radiation treatment, and vascular pathology [10]. Furthermore, these patients were often not great surgical candidates due to their multiple comorbidities [10]. Despite this, open surgery is advantageous in its ability to treat UIAFs in scenarios with a high index of suspicion despite multiple nondiagnostic imaging tests, as proven in our second case [11]. In the current era, endovascular treatment is the preferred choice of intervention due to its minimally invasive nature, as well as its ability to maintain distal perfusion to the pelvis and lower extremities, and has shown to be successful in managing bleeding [2, 11]. Endovascular treatment typically consists of stent grafting with embolization. An early complication rate from endovascular treatment at a single institution was reported at 27%, consisting of graft infection, lower limb ischemia, stent graft thrombosis, and non-ST elevation myocardial infarction [4]. The most serious of which is graft infection, and some even consider infection or an unsterile environment to be a contraindication to endovascular treatment [5]. Unfortunately, due to the rarity and morbidity associated with this condition, the overall efficacy and complication rate are poorly studied. Long-term complications of both treatment modalities include lower extremity ischemia, renal loss, and recurrent hemorrhage [12]. The most common is lower limb ischemia with a prevalence of 67% with open repair vs. 50% from endovascular stenting [12]. Several studies have attempted to compare the outcome and complications between UIAF treatments with no clear advantage [9, 12]. We believe that these cases not only highlight the difficulty with managing this condition but also support endovascular management as the optimal management when feasible. Case 1 highlights successful endovascular management with minimal associated morbidity. Case 2 highlights some of the associated morbidity with an open surgical repair including both lower limb ischemia and increased blood loss. That being said, both approaches ultimately proved successful in the management of a life-threatening condition. Ultimately, it is apparent that treatment of a UIAF requires a multidisciplinary team of urologists, radiologists, and vascular surgeons.
